# Development of a Deep Learning Model to Estimate Anemia from Palpebral Conjunctiva Taken with a Portable Slit-Lamp Microscope

**DOI:** 10.3390/bioengineering13070824

**Published:** 2026-07-17

**Authors:** Yo Nakahara, Eisuke Shimizu, Takahiro Mizukami, Hiroki Nishimura, Shintaro Nakayama, Tetsuo Ishikawa, Masatoshi Hirayama, Risa Hokama, Kazuhiro Sakurada, Kazuno Negishi

**Affiliations:** 1 Department of Extended Intelligence for Medicine, The Ishii-Ishibashi Laboratory, Keio University School of Medicine, Tokyo 1608582, Japan; yo_nakahara@keio.jp (Y.N.); tetsuo.ishikawa@keio.jp (T.I.); kzsakurada@keio.jp (K.S.); 2 Medimo Inc., Tokyo 1050001, Japan; 3 OUI Inc., Tokyo 1070062, Japan; ophthalmolog1st.acek39@keio.jp (E.S.); hiroki@ouiinc.jp (H.N.); p.shintaro@ouiinc.jp (S.N.); 4 Department of Ophthalmology, Keio University School of Medicine, Tokyo 1608582, Japan; mar_hirayama@keio.jp (M.H.); lisayamazaki@keio.jp (R.H.); kazunonegishi@keio.jp (K.N.); 5 Yokohama Keiai Eye Clinic, Yokohama 2400065, Japan; 6 Division of Applied Mathematical Science, RIKEN Center for Interdisciplinary Theoretical and Mathematical Sciences, RIKEN, Yokohama 2300045, Japan; 7 Collective Intelligence Research Laboratory, Graduate School of Arts and Sciences, The University of Tokyo, Tokyo 1538902, Japan; 8 Department of Ophthalmology, Faculty of Medicine, Fukuoka University, Fukuoka 8140180, Japan

**Keywords:** anemia, hemoglobin, palpebral conjunctiva, smart eye camera, smartphone, deep learning, ConvNeXt

## Abstract

**Background:** Anemia is a common systemic condition associated with adverse maternal, perioperative, and cardiovascular outcomes. Although timely screening is clinically important, diagnosis still relies on invasive blood testing. Palpebral conjunctival pallor has traditionally been used as a noninvasive indicator of anemia, but its diagnostic accuracy remains limited. This study aimed to develop and validate a deep learning system to estimate hemoglobin (Hb) concentration and screen for anemia using palpebral conjunctiva images captured with a smartphone-compatible slit-lamp microscope. **Methods:** In this prospective observational study, 225 Japanese participants (20–92 years) underwent conjunctival imaging and blood testing. Palpebral conjunctiva videos were obtained using the Smart Eye Camera. Video frames were processed using automated anterior-segment segmentation and conjunctiva extraction. A ConvNeXt-based regression model was trained to predict Hb values. Anemia was defined using sex-specific Hb thresholds. **Results:** From 225 videos, 53,776 frames were extracted, yielding 9903 quality-filtered conjunctiva images (training: 8082; test: 1821). Video-level predicted Hb values moderately correlated with measured Hb (*r* = 0.42). For anemia screening, frame-level analysis achieved an AUC of 0.75, with accuracy of 0.76, sensitivity of 0.71, and specificity of 0.79. Video-level aggregation achieved 69% accuracy. **Conclusions:** Deep learning analysis of palpebral conjunctiva images acquired with a portable slit-lamp microscope demonstrated the feasibility of non-invasive hemoglobin estimation and anemia screening. Although the proposed approach achieved moderate performance, further improvements in model accuracy and prospective multi-center validation are required before clinical implementation.

## 1. Introduction

Anemia is one of the most prevalent global health conditions, affecting approximately one in four individuals worldwide and leading to substantial reductions in quality of life as well as unfavorable clinical outcomes [1,2,3,4]. In vulnerable populations, unrecognized anemia has been associated with poorer maternal outcomes, including postpartum hemorrhage [5], increased perioperative morbidity and mortality [6], and worse outcomes in chronic cardiovascular disorders such as heart failure [7]. In addition, anemia may represent an early clinical indicator of underlying diseases, including gastrointestinal bleeding [8]. Nevertheless, prompt identification of anemia remains challenging because standard hemoglobin (Hb) assessment relies on blood collection, laboratory equipment, and trained healthcare personnel, making routine screening difficult in settings with limited resources, restricted access, or demanding clinical workflows.

The initial clinical evaluation of suspected anemia has traditionally included assessment of physical findings such as conjunctival pallor [9]. The palpebral conjunctiva is particularly suitable for non-invasive anemia assessment because it is thin, minimally pigmented, and contains a dense superficial capillary network [9]. Reductions in hemoglobin concentration decrease the redness of this highly vascularized tissue, making conjunctival pallor a clinically recognized indicator of anemia. However, the diagnostic performance of pallor-based assessment is inconsistent, with considerable variability in both accuracy and interobserver agreement [9,10]. Noninvasive Hb estimation based on digital analysis of conjunctival appearance has been the focus of numerous studies. Initial investigations employed digital imaging of the palpebral conjunctiva [11], whereas later studies estimated Hb levels by objectively measuring conjunctival pallor from photographs obtained with commercially available cameras [12]. Other optical methods have also been proposed, including the use of conjunctival reflectance spectra to predict Hb concentration [13,14,15].

Recent advances have expanded noninvasive Hb estimation to include smartphone-based imaging and machine learning techniques applied to the fingernail bed [16], fingertip [17], palm, and conjunctiva [18,19]. Although these approaches offer considerable potential as inexpensive screening tools, their performance may be compromised by variations in ambient illumination, differences in camera hardware, and inconsistent image acquisition, often necessitating large training datasets and calibration procedures.

Slit-lamp microscopes are routinely available in ophthalmology practice and provide standardized illumination, a fixed working distance, and magnified visualization of the ocular surface. The Smart Eye Camera (SEC; OUI Inc., Tokyo, Japan), a smartphone-compatible handheld slit-lamp, enables acquisition of slit-lamp–quality anterior segment videos using a compact and portable device and has been clinically validated for anterior segment examination [20,21,22,23,24]. In contrast to conventional smartphone imaging performed under ambient conditions, slit-lamp–based imaging offers more consistent image acquisition by minimizing variations in illumination, camera-to-subject distance, and magnification [20]. We therefore postulated that these advantages would facilitate the extraction of conjunctival vascular features relevant to hemoglobin estimation, improve image consistency, and support efficient deep learning model development using a relatively small training dataset.

Because the performance requirements differ between screening and definitive diagnosis, our primary objective was to develop a non-invasive screening tool that could identify individuals who may benefit from confirmatory blood testing, rather than replace laboratory hemoglobin measurement. In this study, we developed and validated a deep learning model for estimating Hb concentration and identifying anemia using palpebral conjunctival videos acquired with a handheld slit-lamp microscope. We also established an automated preprocessing pipeline for extracting the palpebral conjunctiva from slit-lamp video recordings and assessed model performance at both the frame and video levels.

## 2. Materials and Methods

### 2.1. Study Design and Ethics

This prospective observational study adhered to the tenets of the Declaration of Helsinki and was approved by the institutional review board of Keio University School of Medicine (IRB No. 20200283; Date 1 February 2021). Informed consent was obtained from all subjects involved in the study.

### 2.2. Participants and Reference Standard

Consecutive eligible patients who underwent anterior segment examination with the SEC and had a complete blood count available within ±14 days of imaging were prospectively enrolled. Patients with conditions likely to alter conjunctival color or anatomy (e.g., conjunctival hemorrhage, active conjunctivitis, severe ocular surface disease, or significant motion or defocus precluding region extraction) were excluded. Hemoglobin (Hb) concentration (g/dL) obtained from the complete blood count served as the reference standard. For binary classification, anemia was defined using sex-specific thresholds (men < 13.0 g/dL; women < 12.0 g/dL) [1].

### 2.3. Image Collection with a Portable Slit-Lamp Microscope

Anterior segment videos were obtained using the Smart Eye Camera (SEC; SLM-i07/SLM-i08SE, OUI Inc., Tokyo, Japan), a handheld smartphone-compatible slit-lamp device that enables portable anterior segment imaging [20,21,22,23,24]. Video acquisition was performed with either an iPhone 7 or an iPhone SE (2nd generation) (Apple Inc., Cupertino, CA, USA), producing recordings with resolutions of 720 × 1280 or 1080 × 1920 pixels at frame rates ranging from 30 to 60 frames per second, depending on the imaging configuration. During image acquisition, the lower eyelid was gently everted to expose the palpebral conjunctiva, and videos were recorded under standardized slit-lamp illumination.

### 2.4. Preprocessing: Conjunctiva Segmentation and Cropping

**Figure 1:**
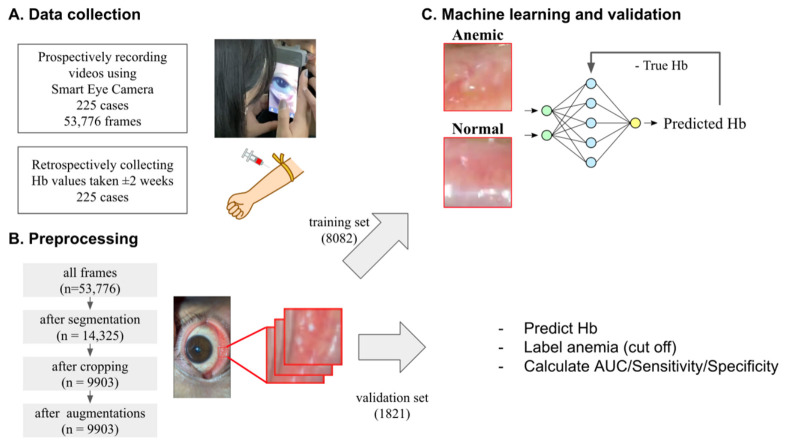
Study design and automated preprocessing pipeline. Palpebral conjunctiva videos were recorded with the Smart Eye Camera (SEC). Frames were extracted (*n* = 53,776), filtered using automated segmentation to retain frames with sufficient palpebral conjunctiva area (*n* = 14,325), and cropped to standardized 100 × 100 conjunctiva regions of interest (*n* = 9903). The dataset was split at the video level into training (8082 frames) and test (1821 frames).

Individual frames were extracted from all recorded videos for subsequent analysis. To minimize interference from surrounding anterior segment structures, an automated segmentation model was used to detect the palpebral conjunctiva. Frames were discarded when the detected conjunctival region was smaller than a predefined area threshold, indicating insufficient conjunctival exposure, or when multiple conjunctival regions were identified. For the retained frames, the centroid of the segmentation mask was calculated, and a fixed 100 × 100-pixel region of interest was cropped around this point. This preprocessing strategy is consistent with previously reported anterior segment image analysis workflows [20,21,22,23,24]. Of the 53,776 extracted frames, 14,325 were retained after segmentation, resulting in 9903 cropped palpebral conjunctiva images following region-of-interest extraction and quality filtering (Figure 1).

### 2.5. Deep Learning Model Development

A regression model was developed to predict Hb concentration from cropped palpebral conjunctiva images. We adopted ConvNeXt (base-384) [25], pretrained on ImageNet, as the feature extraction backbone and replaced its final classification layer with a regression head that generated a single continuous Hb value. ConvNeXt was selected because it has demonstrated strong performance across a wide range of image recognition tasks while maintaining compatibility with ImageNet pretraining, making it suitable for transfer learning on relatively small medical imaging datasets [25]. Model optimization was performed by minimizing the mean squared error between the predicted and reference Hb measurements. To avoid data leakage arising from highly correlated images, the dataset was partitioned at the participant level before model development. Because each participant contributed a single video, all frames derived from the same participant were assigned exclusively to either the training or test set, ensuring complete separation between datasets. The resulting training and test datasets comprised 8082 and 1821 frames, respectively (Figure 1). During training, data augmentation, including random rotation, brightness and contrast adjustment, and image blurring, was applied to improve model robustness. Gradient-weighted Class Activation Mapping (Grad-CAM) was subsequently used to generate heatmaps highlighting image regions that contributed most to the model predictions [26].

### 2.6. Statistical Analysis

Model performance for Hb estimation was assessed by examining the correlation between predicted and measured Hb values. In addition, mean absolute error (MAE), mean absolute percentage error (MAPE), and root mean squared error (RMSE) were calculated to quantify prediction accuracy. To obtain video-level estimates, frame-level predictions were averaged across all frames belonging to the same video. For anemia detection, receiver operating characteristic (ROC) curve analysis was performed using the predicted Hb values as the test variable and anemia status as the reference standard, and the corresponding area under the curve (AUC) was calculated. Diagnostic performance was further evaluated using the confusion matrix, from which accuracy, sensitivity, specificity, positive predictive value (PPV), negative predictive value (NPV), and F1-score were calculated at the predefined anemia threshold. Confidence intervals (CIs) for all performance metrics were estimated by participant-level bootstrap resampling.

## 3. Results

### 3.1. Study Population and Dataset

A total of 225 participants were included. Participants were 20–92 years of age (mean 57.77 ± 16.91 years); 64 were male and 161 were female. Based on sex-specific Hb thresholds, 59 participants met criteria for anemia and 166 did not. All participants were Japanese (Table 1).

From 225 slit-lamp videos, 53,776 frames were extracted. Automated segmentation retained 14,325 frames with adequate palpebral conjunctiva visualization, and automated cropping generated 9903 standardized conjunctiva regions of interest (Figure 1).

### 3.2. Hemoglobin Estimation Performance

**Figure 2:**
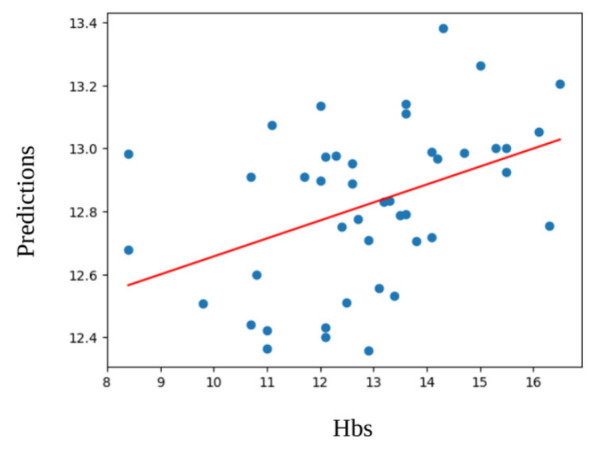
Hemoglobin estimation performance. Scatter plot of predicted versus measured hemoglobin (Hb) in the held-out test set using video-unit aggregation (mean predicted Hb across frames per video). Pearson correlation coefficient *r* = 0.42 (*n* = 44 test videos).

Video-level Hb estimates (mean of frame predictions within each video) showed a moderate correlation with measured Hb (Pearson *r* = 0.42; *n* = 44 test videos). Results are shown in Figure 2. At the frame level, the MAE, MAPE, and RMSE were 1.306 g/dL, 10.464%, and 1.666 g/dL, respectively. At the video level, the corresponding values were 1.375 g/dL, 11.291%, and 1.770 g/dL, respectively.

### 3.3. Anemia Screening Performance

For anemia screening, frame-level predictions in the test dataset achieved an AUC of 0.75, with an accuracy of 0.76 (95% CI, 0.74–0.78), sensitivity of 0.71 (95% CI, 0.68–0.73), specificity of 0.79 (95% CI, 0.76–0.81), positive predictive value of 0.56 (95% CI, 0.52–0.59), negative predictive value of 0.88 (95% CI, 0.86–0.90), and an F1-score of 0.62 (95% CI, 0.60–0.64). The corresponding confusion matrix consisted of 353 true positives, 1038 true negatives, 147 false positives, and 283 false negatives. Video-level aggregation achieved an AUC of 0.69, with an accuracy of 0.69, sensitivity of 0.64, and specificity of 0.73. Because the number of test videos was limited (*n* = 44), a confusion matrix was not generated for the video-level analysis.

### 3.4. Visualization

**Figure 3:**
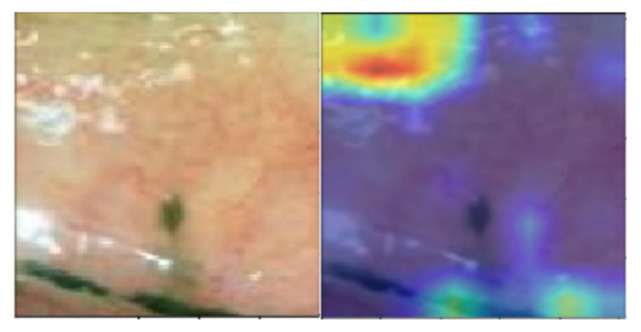
Grad-CAM model interpretability. Representative Grad-CAM heatmap overlaid on a palpebral conjunctiva image illustrating image regions contributing most to hemoglobin estimation. Warmer colors indicate greater contribution to the model output.

A representative Grad-CAM heatmap overlaid on a palpebral conjunctiva image, illustrating regions contributing to hemoglobin estimation, is shown in Figure 3.

### 3.5. Representative Figures

**Figure 4:**
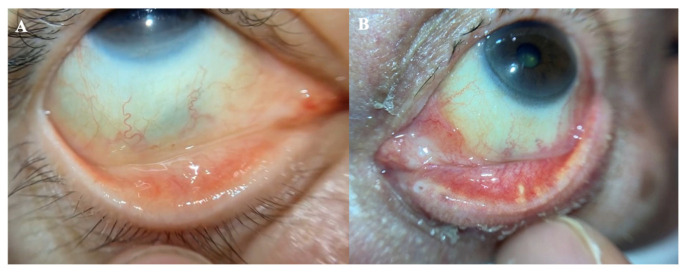
Representative examples of hemoglobin estimation using the proposed deep learning model. (**A**) Representative anemic case with a measured Hb concentration of 9.8 g/dL and a predicted Hb concentration of 9.5 g/dL. (**B**) Representative non-anemic case with a measured Hb concentration of 12.0 g/dL and a predicted Hb concentration of 12.9 g/dL.

Representative examples of model predictions are shown in Figure 4. These examples illustrate the model’s ability to estimate Hb concentration across different clinical presentations of the palpebral conjunctiva.

## 4. Discussion

### 4.1. Principal Findings and Clinical Relevance

In this study, we developed a deep learning system to estimate Hb concentration and screen for anemia from palpebral conjunctiva videos acquired with a portable slit-lamp microscope. The palpebral conjunctiva is routinely examined for pallor in clinical practice, but its interpretation is subjective and may vary depending on examiner experience, lighting conditions, and conjunctival pigmentation. The model achieved a moderate correlation between predicted and measured Hb at the video level (Pearson *r* = 0.42) and demonstrated moderate discrimination for anemia screening (frame-level AUC = 0.75). Our approach converts slit-lamp videos into standardized cropped conjunctival images through automated segmentation, followed by Hb estimation using a ConvNeXt-based regression model. To better understand the practical utility of this approach, we evaluated performance at both the frame and video levels. Although averaging predictions across multiple frames was intended to reduce the influence of motion blur, specular reflection, and partial conjunctival exposure, the observed decrease in AUC suggests that simple averaging may also dilute highly informative frames. In addition, video-level analysis was performed on a substantially smaller number of samples than frame-level analysis, which may have contributed to greater variability in the performance estimates. Future studies should investigate more sophisticated video-level aggregation strategies, such as quality-weighted averaging or attention-based temporal modeling, to better exploit the information contained within video sequences. From a clinical perspective, although the present model demonstrated moderate performance, it is not intended to replace laboratory hemoglobin measurement. Rather, its potential clinical role is as a non-invasive screening or triage tool to identify individuals who may benefit from confirmatory blood testing. Because screening applications generally prioritize high sensitivity to minimize missed cases, further improvements in model performance will be required before clinical implementation. In addition, integration into routine clinical workflows will require prospective validation in diverse populations and evaluation of its impact on clinical decision-making.

### 4.2. Comparison with Prior Conjunctiva-Based Hb/Anemia Estimation

Initial proof-of-concept studies demonstrated that Hb concentration could be estimated from digital images of the palpebral conjunctiva, with a moderate correlation observed between image-derived estimates and measured Hb values (*r* = 0.522) in a prospective validation cohort [11]. Subsequent studies further showed that objective quantification of conjunctival pallor from digital photographs could be used for anemia screening, achieving an AUC of 0.82 with a sensitivity of 84.5% and a specificity of 76.0% for the detection of moderate anemia [12]. More recently, smartphone-based methods have demonstrated favorable diagnostic performance for anemia detection, reporting an accuracy of 72%, sensitivity of 73%, and specificity of 83% [18]. In addition, approaches incorporating RAW image acquisition and real-time processing have achieved excellent discrimination at clinically relevant Hb thresholds, with an AUC of 0.92 for identifying severe anemia (Hb < 7 g/dL) [19].

Direct comparison of reported performance across studies remains challenging because of differences in image acquisition methods, study populations, and evaluation strategies. For example, one study using a relatively small dataset reported only a moderate correlation between convolutional neural network (CNN)-based Hb predictions and laboratory measurements (*r* = 0.44), together with high specificity but limited sensitivity for anemia detection, likely reflecting the effects of both sample size and class imbalance [27]. Other investigators have combined conjunctival images with demographic and anthropometric information, achieving a lower RMSE of 0.68 g/dL in a blood donor population [28]. In contrast, patient-specific models that are fine-tuned for individual subjects have demonstrated substantially improved agreement with reference Hb values (R^2^ = 0.94; MAE, 0.25 g/dL); however, these approaches are primarily designed for longitudinal follow-up rather than population-based screening and are therefore not directly comparable [29]. Furthermore, classification-based models using conjunctival images alone have achieved excellent discrimination in curated datasets, with reported AUC values as high as 0.97 [30].

Table 2 summarizes diagnostic performance at the frame and video levels and provides a comparison with a representative prior smartphone-based conjunctiva approach. Table 3 summarizes representative prior work using palpebral conjunctiva imaging or sensing for Hb estimation.

Against this background, our study introduces a deep learning workflow specifically designed for slit-lamp imaging. The proposed approach incorporates (i) standardized illumination and a fixed working distance, (ii) video-based image acquisition that provides multiple candidate frames from a single examination, and (iii) a fully automated preprocessing pipeline for palpebral conjunctiva localization. Although the predictive performance was moderate, our findings indicate that slit-lamp video imaging may represent a practical intermediate approach between conventional smartphone-based imaging under ambient conditions and dedicated optical spectroscopy systems.

The characteristic pallor of the palpebral conjunctiva observed in anemia is primarily attributable to reduced hemoglobin within the dense superficial capillary plexus of the conjunctival tissue. Owing to its thin, nonkeratinized, and minimally pigmented structure, the palpebral conjunctiva exhibits a noticeable reduction in hemoglobin-dependent red coloration as Hb concentration decreases, resulting in its pale clinical appearance [9]. In agreement with this physiological basis, Grad-CAM analysis showed that the model focused predominantly on the posterior (tarsal) palpebral conjunctiva, which is regarded as the optimal site for clinical assessment of anemia because of its abundant superficial capillary network (Figure 3).

We speculate that slit-lamp–based imaging possesses several characteristics that may improve model development efficiency by reducing the volume of training data required to achieve a given level of performance. Standardized illumination and a fixed working distance help minimize variations in image brightness and color compared with smartphone images acquired under uncontrolled ambient lighting. In addition, the optical magnification provided by the slit-lamp allows finer visualization of conjunctival microvascular and textural features that may reflect tissue Hb status. Furthermore, video acquisition generates multiple frames from a single examination, facilitating automated selection of optimal images and more reliable prediction through video-level aggregation. Collectively, these advantages may have enabled effective training of the regression model using a relatively limited number of high-quality palpebral conjunctival regions of interest. Moreover, data augmentation has become a standard strategy in deep learning–based medical image analysis to improve model generalization when training data are limited. Previous studies have routinely employed augmentation techniques such as image flipping and random cropping to increase the diversity of the training data and improve model robustness [32]. Therefore, the combination of standardized slit-lamp imaging and conventional data augmentation may further contribute to efficient model development, even with relatively modest datasets.

### 4.3. Limitations

This study has several limitations. First, it was conducted in a single-center Japanese population using a single portable slit-lamp device under standardized imaging conditions. Consequently, the generalizability of the proposed model to other ethnicities, conjunctival pigmentation levels, imaging devices, lighting conditions, and clinical environments remains uncertain. External validation using independent multi-center datasets with diverse populations and imaging conditions will therefore be essential to establish the robustness and generalizability of the proposed system. Second, Hb values were obtained from routine clinical blood tests and were not always measured on the same day as imaging, which may have attenuated the observed correlation. Future prospective studies should strengthen the reference standard by minimizing the interval between imaging and blood sampling, ideally using same-day measurements or a narrower time window (e.g., within ±7 days). Third, regression outputs showed compression toward the mean Hb, suggesting that further improvements in model calibration and a broader distribution of Hb values in the training dataset may improve predictive accuracy. Fourth, because this was a retrospective study based on routinely collected clinical data, comprehensive information regarding systemic and ocular comorbidities that might influence conjunctival appearance was not consistently available. Furthermore, although participant-level separation successfully prevented data leakage, multiple frames extracted from the same participant remained highly correlated, meaning that the effective diversity of the dataset was determined by the number of participants rather than the number of images. Although ImageNet pretraining and data augmentation improved learning efficiency, the overall sample size remained relatively modest for a ConvNeXt-base model, limiting the robustness of model development and subgroup analyses according to hemoglobin level, anemia severity, age, sex, and image quality. Larger prospective cohorts will be required to evaluate potential systematic biases and improve model generalizability. Finally, although ConvNeXt was selected because of its strong image recognition performance and compatibility with ImageNet pretraining, we did not compare its performance with alternative convolutional or transformer-based architectures, such as ResNet, EfficientNet, Vision Transformer, or Swin Transformer. Similarly, ablation studies were not performed to quantify the contributions of preprocessing, conjunctiva segmentation, image cropping, data augmentation, or video-level aggregation. Future work should systematically compare different model architectures, perform ablation analyses, and investigate more advanced video-based spatiotemporal modeling rather than simple frame-level averaging to further optimize the proposed framework. Clinically relevant operating points should also be evaluated with confidence intervals in larger prospective cohorts.

### 4.4. Future Directions

Recent advances in deep learning have demonstrated that clinically meaningful information can be extracted from standardized medical images across diverse modalities, including dermoscopic skin images and gastrointestinal endoscopic images [33,34]. These studies highlight the ability of deep learning to identify subtle image features that are difficult to recognize visually, providing a conceptual foundation for non-invasive hemoglobin estimation from conjunctival images. In addition to conjunctival imaging, deep learning–based approaches have recently been applied to posterior segment images, including ultra-widefield and conventional retinal fundus photographs, for anemia prediction [35,36]. These findings collectively suggest that ophthalmic images acquired during routine clinical practice may serve as noninvasive biomarkers reflecting systemic hematologic status. Furthermore, recent review articles have emphasized the growing application of artificial intelligence for anemia screening across a range of imaging and sensing modalities [37], including techniques based on conjunctival and fingernail images [30,38]. Our previous work demonstrated that appropriate image enhancement improves the visibility of anterior segment structures and increases the diagnostic reliability of portable slit-lamp imaging, although excessive enhancement may degrade clinically relevant information [39]. Given these findings, future studies should investigate whether optimized image enhancement or image preprocessing can improve the performance of deep learning models for hemoglobin estimation from conjunctival images.

## 5. Conclusions

A deep learning system using palpebral conjunctiva videos acquired with a portable slit-lamp microscope demonstrated the feasibility of non-invasive hemoglobin estimation and anemia screening. Although the proposed approach achieved moderate performance, further improvements in model accuracy, larger multi-center datasets, and prospective external validation are required before clinical implementation. Standardized slit-lamp imaging combined with automated image analysis represents a promising platform for future non-invasive anemia screening.

## 6. Patents

OUI Inc. has the patent for the Smart Eye Camera and relations (Patent No. JP; 6627071, USA; 12,193,745, EU; 19743494.7, China; 201980010174-7, India; 541687, VN; 1-2020-04893, and Africa; AP6569, AP6839. Patent pending JP; 2019-198354, 2021-535472, 2022-500489, 2024-205339. EU; 20846667-2, 2175926.2, US; 17/799043, VN; 1-2022-01003, Africa; AP/P/2022/013851). There are no other relevant declarations relating to these patents.

## Figures and Tables

**Table 1:** Participant characteristics.

Characteristic	
Participants, *n*	225
Age, years	57.77 ± 16.91 (range 20–92)
Sex, *n* (%)	Male 64 (28.4%); Female 161 (71.6%)
Anemia, *n* (%)	59 (26.2%)
Non-anemia, *n* (%)	166 (73.8%)
Ethnicity	Japanese (100%)

**Table 2:** Representative prior works on diagnostic performance for anemia screening.

Study	Unit of Analysis	Accuracy	Sensitivity	Specificity	AUC
Our study	Video-unit	0.69	0.64	0.73	0.69
Our study	Frame-unit	0.76	0.71	0.79	0.75
Collings et al. 2016 [12]	Conjunctiva photos (consumer camera/smartphone; ambient light)	NA	0.85	0.76	0.82
Suner et al. 2021 [18]	Patient-unit (smartphone conjunctiva)	0.72	0.73	0.83	NA
Sehar et al. 2025 [30]	Palpebral conjunctiva smartphone images	NA	NA	NA	0.97

AUC, Area under the curve; NA, Not applicable.

**Table 3:** Representative prior works on diagnostic performance for hemoglobin estimation.

Study	Input	Population/Setting	Key Performance
Suner et al. 2007 [11]	Palpebral conjunctiva photos (digital camera)	Emergency department; *n* = 65 (44 derivation, 19 evaluation)	Correlation with measured Hb: *r* = 0.634 (derivation), *r* = 0.522 (evaluation).
Kim et al. 2014 [14]	Palpebral conjunctiva reflectance spectroscopy	Spectroscopy + stochastic modeling	Reported sensitivity 86% for clinically diagnosed anemia cases.
Çuvadar et al. 2023 [28]	Conjunctival images + demographic/anthropometric variables	Blood donor cohort (Turkish Red Crescent)	Bias 0.26 g/dL; RMSE 0.68 g/dL; MAPE 3.4%; limits of agreement 1.23 g/dL.
Dimauro et al. 2018 [31]	Conjunctival images (device-assisted)	Device-assisted imaging	Reported feasibility of a non-invasive device and algorithm for conjunctiva-based Hb estimation (performance metrics vary by setting).
Suner et al. 2021 [18]	Palpebral conjunctiva smartphone photos	Smartphone-based point-of-care workflow	Anemia prediction: 72% accuracy, 73% sensitivity, 83% specificity.
Zhao et al. 2024 [19]	Palpebral conjunctiva smartphone RAW images	Real-time smartphone implementation (RAW capture)	Bland–Altman bias 0.10 g/dL (LOA −4.73 to 4.93); AUC 0.92 at Hb = 7 g/dL, 0.90 at 9 g/dL.
Kato et al. 2024 [27]	Palpebral conjunctiva smartphone photos	*n* = 150 images; 10 with Hb < 11 g/dL	Correlation *r* = 0.44 (CNN); AUC 0.82 (non-CNN); sensitivity 20%, specificity 99% for anemia.
Camporeale et al. 2025 [29]	Palpebral conjunctiva images	Patient-specific fine-tuning; repeated measures	R^2^ 0.94; MAE 0.25 g/dL; accuracy 98%; sensitivity 100%.

AUC, Area under the curve; CNN, Convolutional neural network; Hb, Hemoglobin; LOA, Limits of Agreement; MAE, Mean absolute error; MAPE, Mean absolute percentage error; RMSE, Root mean squared error.

## Data Availability

The data presented in this study are not publicly available due to privacy and ethical restrictions. Data may be made available from the corresponding author upon reasonable request.

## Abbreviations

The following abbreviations are used in this manuscript:

AUC	Area under the curve
CNN	Convolutional neural network
Hb	Hemoglobin
LOA	Limits of Agreement
MAE	Mean absolute error
MAPE	Mean absolute percentage error
NA	Not applicable
RMSE	Root mean squared error
ROC	Receiver operating characteristic
SEC	Smart Eye Camera
